# Supplementary material for: The impact of an eco-score label on US consumers’ perceptions of environmental sustainability and intentions to purchase food: A randomized experiment
Source: PLoS One. 2024 Jun 27;19(6):e0306123. doi: 10.1371/journal.pone.0306123 (PMC11210794; doi:10.1371/journal.pone.0306123)
Supplement: S1 Table — (DOCX) [file pone.0306123.s001.docx]

|  | Perceived sustainability | | | | Purchase intentions | | | |
| --- | --- | --- | --- | --- | --- | --- | --- | --- |
|  | Control | | Eco-score label | | Control | | Eco-score label | |
| Burger |  |  |  |  |  |  |  |  |
| Low score | 3.2 | (1.1) | 2.8 | (1.4) | 2.9 | (1.3) | 2.7 | (1.4) |
| High score | 3.5 | (1.1) | 4.0 | (1.1) | 2.5 | (1.4) | 2.8 | (1.4) |
| Pizza |  |  |  |  |  |  |  |  |
| Low score | 3.1 | (1.1) | 2.8 | (1.5) | 3.4 | (1.3) | 3.1 | (1.3) |
| High score | 3.6 | (1.0) | 4.1 | (1.0) | 2.8 | (1.4) | 3.1 | (1.4) |
| Sandwich |  |  |  |  |  |  |  |  |
| Low score | 3.3 | (1.1) | 2.8 | (1.4) | 3.0 | (1.3) | 2.8 | (1.3) |
| High score | 3.4 | (1.0) | 4.0 | (1.0) | 3.0 | (1.3) | 3.2 | (1.2) |
| Snack |  |  |  |  |  |  |  |  |
| Low score | 3.0 | (1.1) | 2.7 | (1.5) | 3.0 | (1.4) | 3.0 | (1.4) |
| High score | 3.6 | (1.1) | 4.2 | (1.0) | 3.2 | (1.3) | 3.5 | (1.3) |
| Total |  |  |  |  |  |  |  |  |
| Low score | 3.2 | (1.1) | 2.8 | (1.4) | 3.1 | (1.3) | 2.9 | (1.3) |
| High score | 3.5 | (1.1) | 4.1 | (1.0) | 2.9 | (1.4) | 3.1 | (1.3) |
| N (observations) | 4,024 |  | 4,080 |  | 4,024 |  | 4,079 |  |
| N (respondents) | 503 |  | 510 |  | 503 |  | 510 |  |
