# Supplementary material for: The impact of an eco-score label on US consumers’ perceptions of environmental sustainability and intentions to purchase food: A randomized experiment
Source: PLoS One. 2024 Jun 27;19(6):e0306123. doi: 10.1371/journal.pone.0306123 (PMC11210794; doi:10.1371/journal.pone.0306123)
Supplement: Attachment — Submitted filename: Response to Reviewers_PLOSONE.docx [file pone.0306123.s002.docx]

**Manuscript Reference Number:** PONE-D-24-04592

**Title:** The impact of an eco-score label on US consumers’ perceptions of environmental sustainability and intentions to purchase food: A randomized experiment

| **#** | **Reviewers’ comments** | **Revision or Response** | **Line Number** |
| --- | --- | --- | --- |
|  | **Academic Editors** |  |  |
| 1 | The paper should be restructured by highlighting problem statement, contribution and significance of the study and justify the methodology. | We have done this by making edits in the introduction and methodology. Please see a more detailed response outlining the changes in Comment #6. | 94-105  119-122 |
|  | **Journal Requirements** |  |  |
| 2 | Please ensure that your manuscript meets PLOS ONE's style requirements, including those for file naming. | We have done this. | N/A |
| 3 | When completing the data availability statement of the submission form, you indicated that you will make your data available on acceptance. We strongly recommend all authors decide on a data sharing plan before acceptance, as the process can be lengthy and hold up publication timelines. Please note that, though access restrictions are acceptable now, your entire data will need to be made freely accessible if your manuscript is accepted for publication. This policy applies to all data except where public deposition would breach compliance with the protocol approved by your research ethics board. If you are unable to adhere to our open data policy, please kindly revise your statement to explain your reasoning and we will seek the editor's input on an exemption. Please be assured that, once you have provided your new statement, the assessment of your exemption will not hold up the peer review process. | We have published the dataset to the Open Science Framework Repository. We have updated the data availability statement in the submission form. | 116-117 |
| 4 | Please review your reference list to ensure that it is complete and correct. If you have cited papers that have been retracted, please include the rationale for doing so in the manuscript text, or remove these references and replace them with relevant current references. Any changes to the reference list should be mentioned in the rebuttal letter that accompanies your revised manuscript. If you need to cite a retracted article, indicate the article’s retracted status in the References list and also include a citation and full reference for the retraction notice. | We have done this. | 371-514 |
|  | **Reviewer #1** |  |  |
| 5 | Good work, the overall article is very well written and analysed. | Thank you. | N/A |
|  | **Reviewer #2** |  |  |
| 6 | Problem statement need to be more rigor and research gaps should be justified. Please mention justification to consider the current population and sample of study. Need justification to use Qualtrics Market Research Survey Panel. Statistical analysis seem ok. While discussion need improvement. Conclusion is very limited need to be comprehensive and precise. | Thanks for these comments. We have addressed these issues in the following ways:   - In the introduction, we now clearly spell out the problem statement in the last paragraph. - In the introduction, we have added a paragraph justifying the population (US adults). - In the methods, we added justification of the study sample as well as justification for using Qualtrics Market Survey Research Panel. - We have re-organized the discussion section to flow more naturally (i.e., including policy implications towards the end of the discussion). We also updated and expanded the discussion to include a broader discussion of policy implications, additional recent literature on this topic, and future research directions. - We have strengthened the conclusion paragraph, including restating the problem statement, discussing policy implications, and noting limitations. | 94-105  119-122  325-346  356-366 |
